# Relationship between size and other radiological features with malignancy in pulmonary nodules; follow-up or pathological diagnosis?

**DOI:** 10.1097/MD.0000000000041823

**Published:** 2025-03-14

**Authors:** Ayla Turkar, Elcin Ersoz Kose

**Affiliations:** a Radiology Department, Sureyyapasa Chest Diseases and Thoracic Surgery Training and Research Hospital, Istanbul, Turkey; b Umraniye Training and Research Hospital, Istanbul, Turkey; c Thoracic Surgery Department, Sureyyapasa Chest Diseases and Thoracic Surgery Training and Research Hospital, Istanbul, Turkey.

**Keywords:** computed tomography, lung cancer, nodule

## Abstract

Pulmonary lesions can be detected even at a few millimeters in size, allowing for detailed assessment of their radiological features. This study aims to determine the most appropriate approach for nodules detected by computed tomography. A total of 526 patients, who underwent surgery for pulmonary nodules or masses and had pathological diagnoses, were included in the study. Demographic features, clinical history, and surgery-related data of the patients were assessed by a thoracic surgeon, whereas radiological features were evaluated by a radiologist. Of the patients, 147 were female and 379 were male. The mean age was 63 years (min 15, max 89), and the average lesion size was 22 mm (min 4, max 116). Postoperative analysis revealed 132 benign lesions (25.1%), 380 malignant (72.2%), and 14 metastases (2.7%). Among 347 patients, the nodule size was below 30 mm. Malignant nodules showed a higher median age and larger lesion size (*P* < .05 for both). Lesion contour, calcification, pleural tail, changes in lesion during follow-up, presence of emphysema, enlarged lymph nodes, history of malignancy, and smoking were statistically significant in determining the nature of the detected lesion. The clinical and radiological characteristics of patients can be utilized to determine the risk of malignancy in detected nodules. Even if the nodule size is small, histopathological diagnosis may be a more suitable option for high-risk patients instead of radiological follow-up.

## 1. Introduction

As access to radiological imaging methods has become more common and image quality has improved, even very small lung lesions can now be detected. Lesions that are not visible on a chest X-ray can be readily identified using computed tomography (CT). Generally, lesions under 30 mm are referred to as nodules, whereas those exceeding 30 mm are categorized as masses.^[1]^ The Fleischner Society’s guidelines for pulmonary nodules are commonly used in the follow-up of these nodules. However, patients under 35 years of age, those with a known history of lung or extrapulmonary cancers, and those who are immunocompromised are not included in this protocol. Additionally, the British Thoracic Society (BTS) and the American College of Chest Physicians (ACCP) offer comprehensive follow-up protocols as well.^[2–4]^

## 2. Material and methods

### 2.1. Study design

In this single-center study, we retrospectively reviewed the medical records of patients who underwent surgical resection for pulmonary nodules or masses in our hospital’s thoracic surgery clinic between 2019 and 2023. It was conducted in accordance with the Declaration of Helsinki. Since the study is retrospective, there is no requirement for an informed consent form. Ethics committee approval was obtained from our hospital’s ethics committee with protocol code 116.2017.*R*-277.

### 2.2. Study setting and participants

A total of 526 patients, who had pathological diagnoses and nodules/masses detected in CT accessible from the hospital’s Picture Archiving and Communication Systems, were included in the study. All patients underwent surgical resection in our hospital’s thoracic surgery clinic for pulmonary nodules and/or masses. The diagnosis of lesions was made via wedge biopsy, segmentectomy, lobectomy, or pneumonectomy. Wedge resection was initially performed on patients, and frozen sections were applied during the operation for diagnosis. If the frozen result indicated benignity, mediastinal lymph node sampling was not performed, and the surgery was terminated. If the frozen result indicated malignancy, the operation continued in the same session, including lung resection and systemic lymph node sampling. Systematic nodal dissection criteria required the removal of at least 6 lymph nodes from at least 3 mediastinal stations, including 1 subcarinal. All specimens were then sent to pathology with formalin fixation for final diagnosis.

Patients diagnosed by needle biopsies but whose macroscopic lesions could not be verified through surgery in our hospital were excluded from the study.

The demographic features, clinical history, and surgical data of the patients were evaluated by a thoracic surgeon, whereas radiological features were evaluated by a radiologist.

Evaluated demographic features and clinical data:

Age
Gender
Smoking history
History of other malignancies
History of other diseases
History of tuberculosis
History of COVID-19
Family history of cancer
Pathological diagnosis of the lesion

Radiological parameters evaluated for the lesion:

Lesion size
Lesion location
Contour
Cavitation
Necrosis
Calcification
Pleural tail
Ground-glass opacity around the lesion
Changes in the lesion during follow-up
Presence of other nodules
Emphysema
Presence of pleural effusion on the same side as the lesion
Presence of enlarged lymph nodes in the mediastinal area (short axis > 1 cm)

Thoracic CTs initially taken in our hospital were mostly non-contrast. Dynamic imaging allowing evaluation of both non-contrast and contrast-enhanced series simultaneously was not routinely performed, so the post-contrast density increase of the lesions could not be assessed.

In patients under the age of 35, the risk of malignancy in detected nodules was very low, so the age threshold was set at 35 years. Two analyses were performed, 1 excluding and 1 including 8 patients under 35 years old.

The smoking history of 79 patients could not be obtained. The remaining 447 patients were classified as nonsmokers, ex-smokers, and active smokers. Sensitivity analysis was performed by including patients with unknown smoking history separately in the nonsmoker, ex-smoker, and active smoker groups, and also by excluding these patients. Thus, the effect of missing data on the differentiation between benign and malignant lesions was assessed. Active smokers were further categorized based on pack years (p/y): 0 to 9, 10 to 19, 20 to 29, and ≥30 p/y. Idiopathic pulmonary fibrosis (IPF) was specifically considered among comorbidities.

Radiological evaluations were conducted using CT images by a thoracic radiologist with 6 years of experience. The longest axis in axial or coronal planes was considered for lesion size. Lesion contours and appearance were categorized as round, oval, lobulated, spiculated, tubular, and ground-glass. Necrosis was radiologically assessed by the presence of visible macro areas of lower density or micro areas creating heterogeneous appearances within the lesion. If the pathology report indicated necrosis but no radiological evidence was observed, radiological findings were considered. If a patient had multiple CTs, lesions were classified as new, progressive, regressive, or stable. The number and location of additional nodules, and whether they exceeded 10 mm, were noted.

Lesions were also analyzed in a subgroup where only those <30 mm were included. Since PET-CT is not routinely performed on every preoperative patient in our hospital, radiological features from PET-CT images were not used in this study.

## 3. Results

A total of 526 patients participated in the study, of whom 147 were female (27.9%) and 379 were male (72.1%). The mean age was 61.89 ± 10.72 years, and the median age was 63 (range 15 to 89). The mean lesion size was 27.53 ± 18.43 mm, and the median lesion size was 22 mm (range 4 to 116). The demographic data and lesion characteristics of the patients are presented in Tables 1 and 2. Post-examination reported 132 benign lesions (25.1%), 380 malignant lesions (72.2%), and 14 metastases (2.7%).

**Table 1:** Demographic data of patients.

	*n* (%)
Gender	
Female	147 (27.9)
Male	379 (72.1)
Age (categorical)	
≤35	8 (1.5)
>35	518 (98.5)
Emphysema	
No	261 (49.6)
Yes	265 (50.4)
Known presence of malignancy	
No	451 (85.7)
Yes	75 (14.3)
Smoking	
No	27 (5.1)
Yes	253 (48.1)
Ex-smoker	167 (31.7)
Not known	79 (15)
Pack years of smoking	
No	97 (18.4)
0 to 9 p/y	40 (7.6)
10 to 19 p/y	65 (12.4)
20 to 29 p/y	68 (12.9)
≥30 p/y	247 (47)
Not known	9 (1.7)
Comorbidity	
No	519 (98.7)
Yes	7 (1.3)
Family history of cancer	
No	492 (93.5)
Yes	34 (6.5)
Tuberculosis	
No	500 (95.1)
Yes	26 (4.9)
COVID history	
No	427 (81.2)
Yes	99 (18.8)

p/y = pack years of smoking.

**Table 2:** Lesion features and radiological findings.

	n (%)
Lesion size (cathegorical)	
≤30 mm	358 (68.1)
>30 mm	168 (31.9)
Lesion location	
Right upper lobe	180 (34.2)
Right middle lobe	25 (4.8)
Right lower lobe	94 (17.9)
Left upper lobe	126 (24)
Left middle lobe	101 (19.2)
Longest axis	
Transverse	246 (46.8)
Anteroposterior	155 (29.5)
Longitudinal	125 (23.8)
Contour and appearance	
Round-oval	77 (14.6)
Lobulated	345 (65.6)
Spiculated	72 (13.7)
Tubulary	4 (0.8)
Ground glass	28 (5.3)
Cavitation	
No	478 (90.9)
Yes	48 (9.1)
Necrosis	
No	487 (93.1)
Yes	36 (6.9)
Calcification	
No	490 (93.2)
Yes	36 (6.8)
Pleural tail	
No	338 (64.3)
Yes	188 (35.7)
Ground glass around the lesion	
No	414 (78.7)
Yes	112 (21.3)
Screening feature	
First scan	376 (71.5)
Newly emerging	38 (7.2)
Progressed	79 (15)
Regressed	6 (1.1)
Stabile	27 (5.1)
Are there any other nodules?	
No	409 (77.8)
Yes	117 (22.2)
If so, how many?	
Only 1 other	43 (36.8)
More than 1	74 (63.2)
If so, is it >1 cm	
No	64 (54.7)
Yes	53 (45.3)
If so, is it in the same lobe?	
No	36 (31.3)
Yes	26 (22.6)
Both in the same and different lobes	53 (46.1)
Ipsilateral pleural effusion	
No	502 (95.4)
Yes	24 (4.6)
Mediastinal lymph node >1cm	
No	497 (94.5)
Yes	29 (5.5)

**Figure 1.**
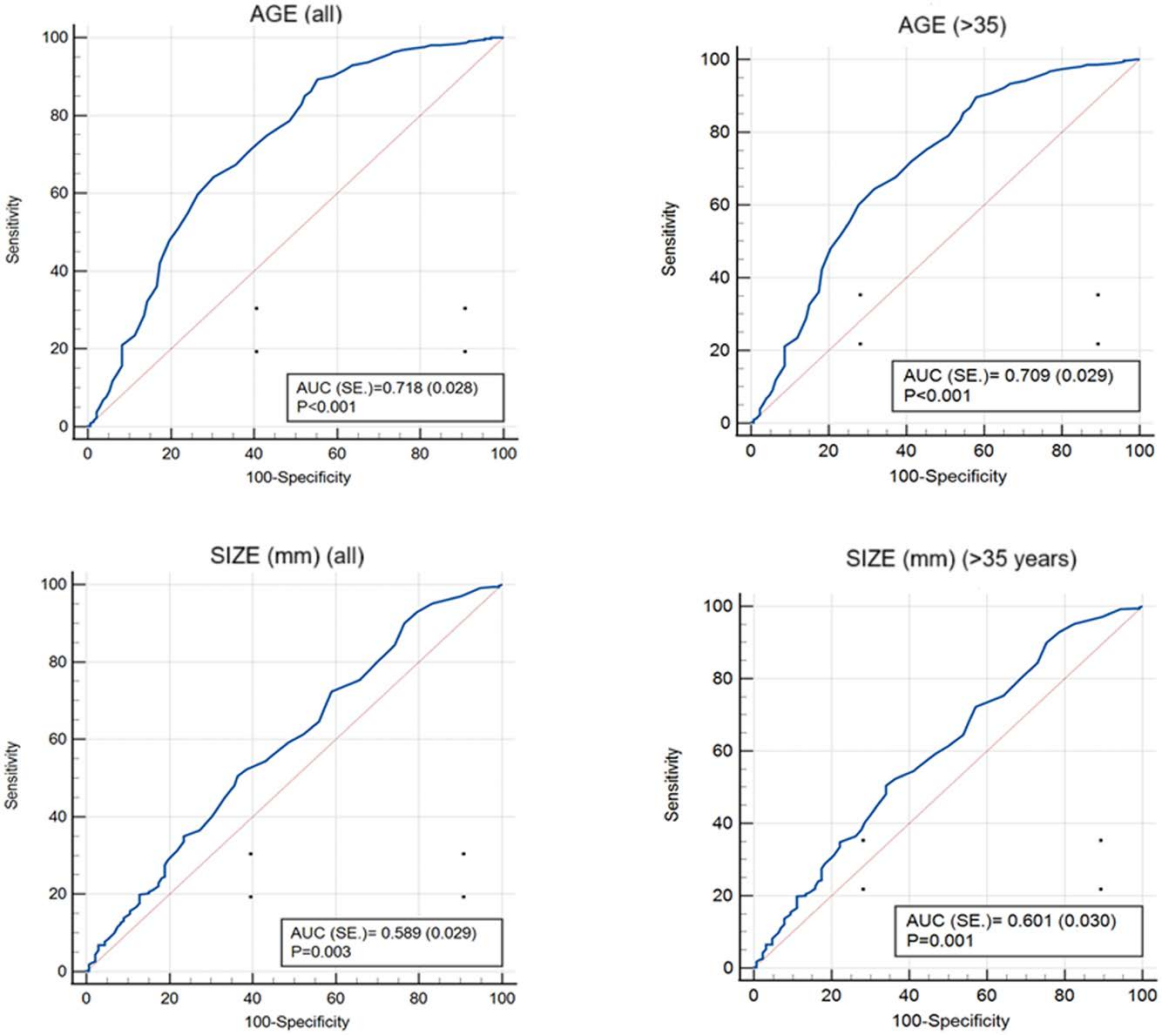
Age and size characteristics of malignant lesions.

In malignant nodules and metastatic lesions, patient age and lesion size were higher compared to the benign group. The risk of malignancy for lesions was significantly higher when patient age exceeded 53 years. When only patients over 35 years old were analyzed, 61 years was identified as the cutoff value (*P* < .001 for both). For lesion size, 23 mm was determined as the threshold. Accordingly, the probability of malignancy was significantly higher for lesions with a maximum axis exceeding 23 mm, both for all patients and for those over 35 years old (*P* = .003 and *P* = .001; Table 3 and Fig. 1).

**Table 3:** Age and size characteristics of malignant lesions.

Reference malignant		Cut off	Sensitivity	Specificity	+PV	−PV	AUC ± SE.	*P* value
Total (n = 526)	Age	>53	89.21	44.7	82.3	59	0.718 ± 0.028	**<.001**
	Lesion size (mm)	>23	50.53	63.64	37.9	98.2	0.589 ± 0.029	**.003**
	Lesion size (mm)	>30*	35	76.2	81.1	29		
Age > 35 (n = 518)	Age	>61	64.55	68.25	85.9	39.1	0.709 ± 0.029	**<.001**
	Lesion size (mm)	>23	50.53	65.87	81,6	30,7	0.601 ± 0.030	**.001**
	Lesion size (mm)	>30*	34.92	77.78	82.5	28.5		

ROC Analysis (Honley&Mc Nell - Youden index J).

The *P* values written in bold are intended to highlight the statistically significant results of the analysis.

AUC = area under the ROC curve; +PV = positive predictive value; −PV = negative predictive value; ROC = receiver operating curve; SE = standard error.

* Cut off value for nodule and mass distinguishing

**Figure 2.**
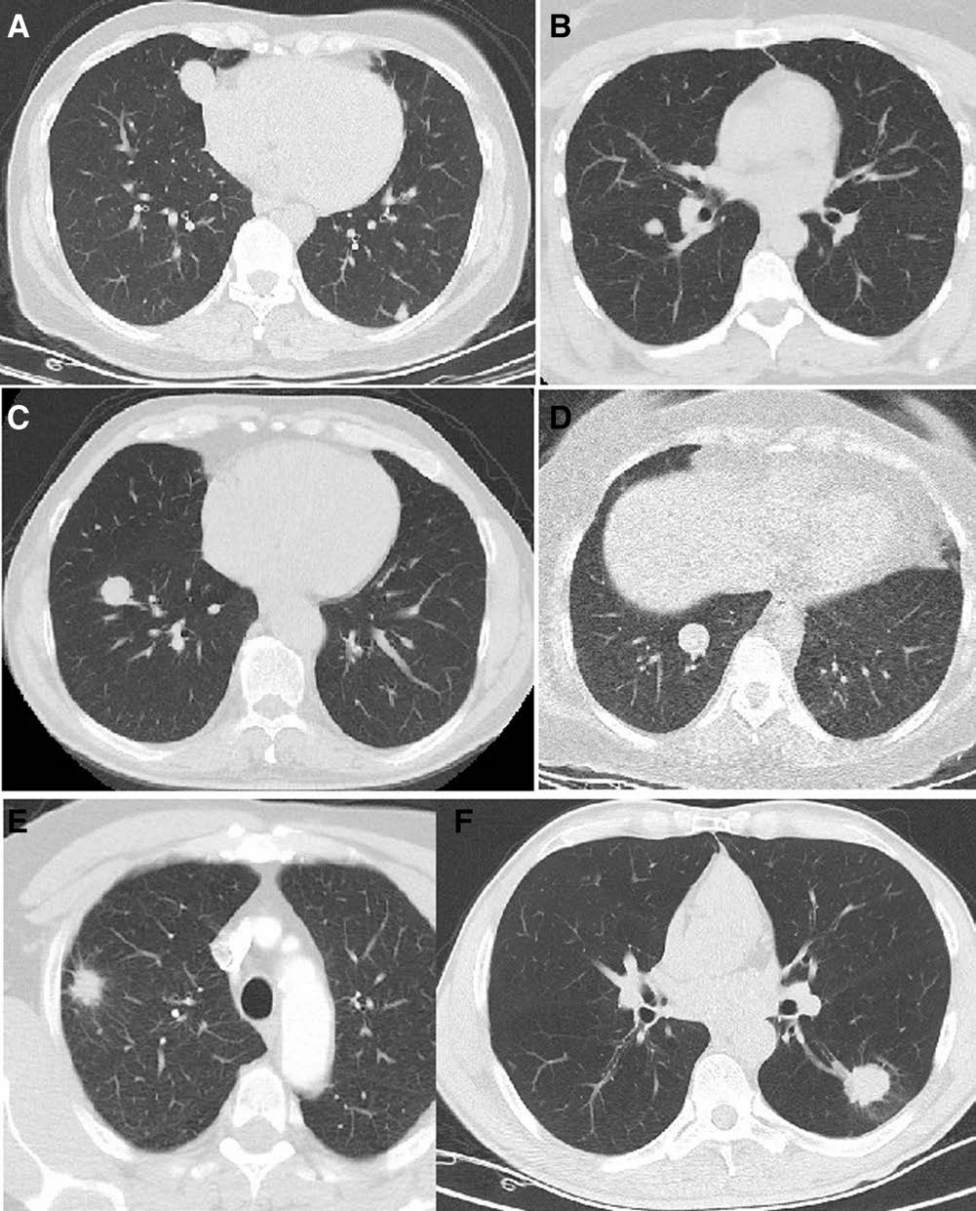
(A) cavernous hemangioma-arteriovenous malformation, (B) hamartoma, (C) metastasis of colon carcinoma, (D) carcinoid tumor, (E) squamous cell carcinoma, (F) small cell carcinoma.

Lesion contour also showed differences between the groups. Benign lesions tended to be well-defined, whereas lobulated and spiculated contours were more common in the malignant group. However, metastatic lesions in the malignant group were an exception and had contour characteristics similar to benign lesions (Fig. 2).

Calcification was more frequent in benign lesions (*P* = .003), whereas pleural tail was more commonly associated with malignant lesions (*P* = .002).

**Figure 3.**
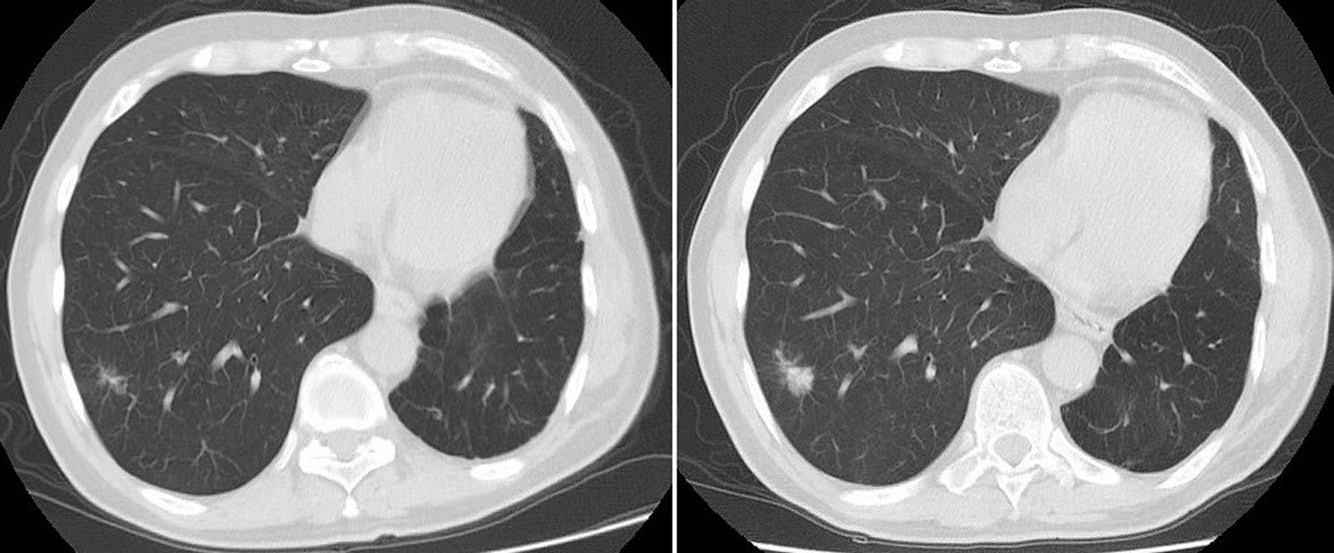
Fifty-nine year-old male patient. The pathological diagnosis of the lesion with progression on follow-up imaging in the lower lobe of the right lung was squamous cell carcinoma. Additionally, this patient had a previous history of left lower lobectomy due to lung cancer.

Lesions that did not show size progression during follow-up were more likely to be benign (*P* < .001), while those showing progression were more likely to be malignant (*P* = .002; Fig. 3). None of the malignant lesions showed regression during follow-up, while 6 benign lesions (4.5%) regressed.

The presence of other nodules did not statistically differ between benign and malignant groups (*P* = .70); however, when the malignant group was divided into primary and metastatic subgroups, nodules accompanying metastatic lesions were more likely than those accompanying primary lung malignancies (*P* = .033). Additionally, nodules accompanying benign lesions were more common than those with primary malignancies (*P* = .040).

A significant relationship was found between the presence of emphysema and the nature of the lesion. Emphysema was more common in malignant lesions (*P* < .001). Emphysema was also more frequently associated with primary lung malignancies compared to metastatic lesions (*P* = .001). Even in benign lesions, emphysema was more common compared to metastatic lesions (30.3% vs. 14.3%, respectively).

Enlarged lymph nodes (>1 cm in short axis) in the mediastinal area were observed in only one benign lesion, while none of the metastatic lesions had lymph nodes >1 cm. In contrast, 28 primary malignant cases (7.1%) showed enlarged mediastinal lymph nodes (*P* = .008).

Patients with a known history of cancer had a statistically higher likelihood of the detected lesion being malignant (*P* = .001). Moreover, the probability of the lesion being metastatic was greater than the likelihood of it being a primary lung malignancy (*P* < .001).

No significant difference was found in the relationship between smoking and the nature of nodules (*P* = .141); however, when smoking history exceeded 30 pack years, the probability of the detected nodule being a primary lung malignancy was higher than that of it being benign or metastatic (*P* < .001 and *P* = .003, respectively). In the sensitivity analysis conducted to assess the impact of the group with unknown smoking history on the results, when only patients in this group were considered as nonsmokers, the results were statistically significant (*P* = .005), and the likelihood of lesions being benign increased compared to the analysis where all subgroups were evaluated separately.

No statistically significant relationship was found between the nature of the lesion and comorbidities other than cancer, chronic obstructive pulmonary disease (COPD) and IPF, such as family history of cancer, previous tuberculosis, or COVID-19 infection (*P* > .05). The nodules detected in 4 patients previously diagnosed with IPF were all malignant.

**Figure 4.**
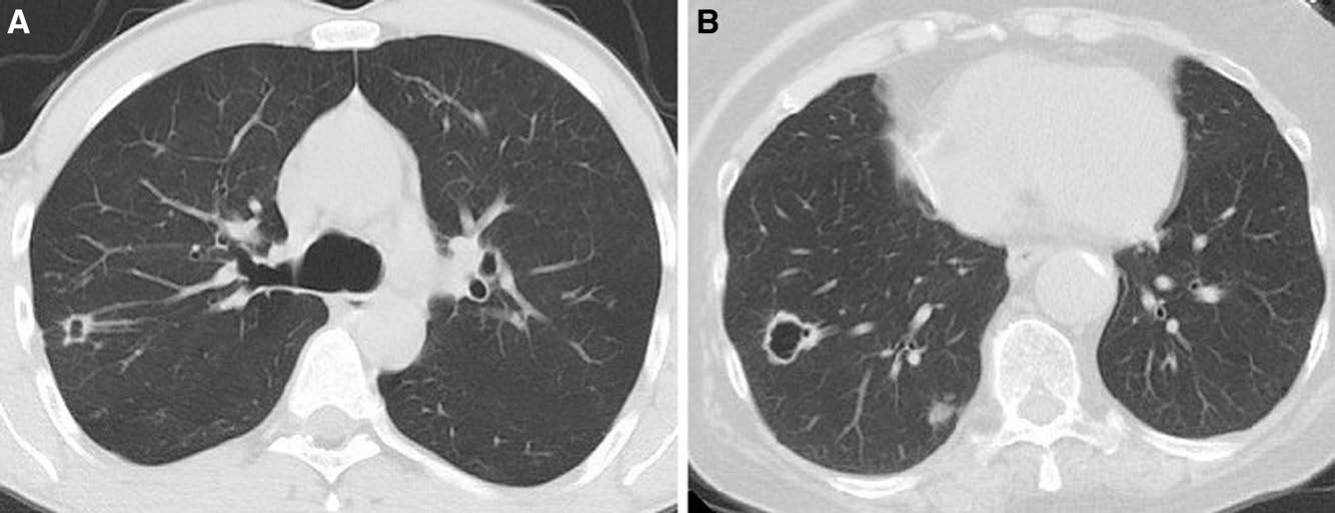
Examples of cavitary lesions, (A) pathological diagnosis is inflammation, (B) squamous cell carcinoma, is also accompanied by other nodules in the same lobe.

Cavitation was found in 9.1% of benign lesions and 9.5% of primary lung malignancies, but in none of the metastatic lesions (*P* = .778; Fig. 4). Similarly, necrosis was observed in 3.9% of benign lesions and 8.2% of primary lung malignancies but was absent in metastatic lesions (*P* = .310; Table 4).

**Table 4:**
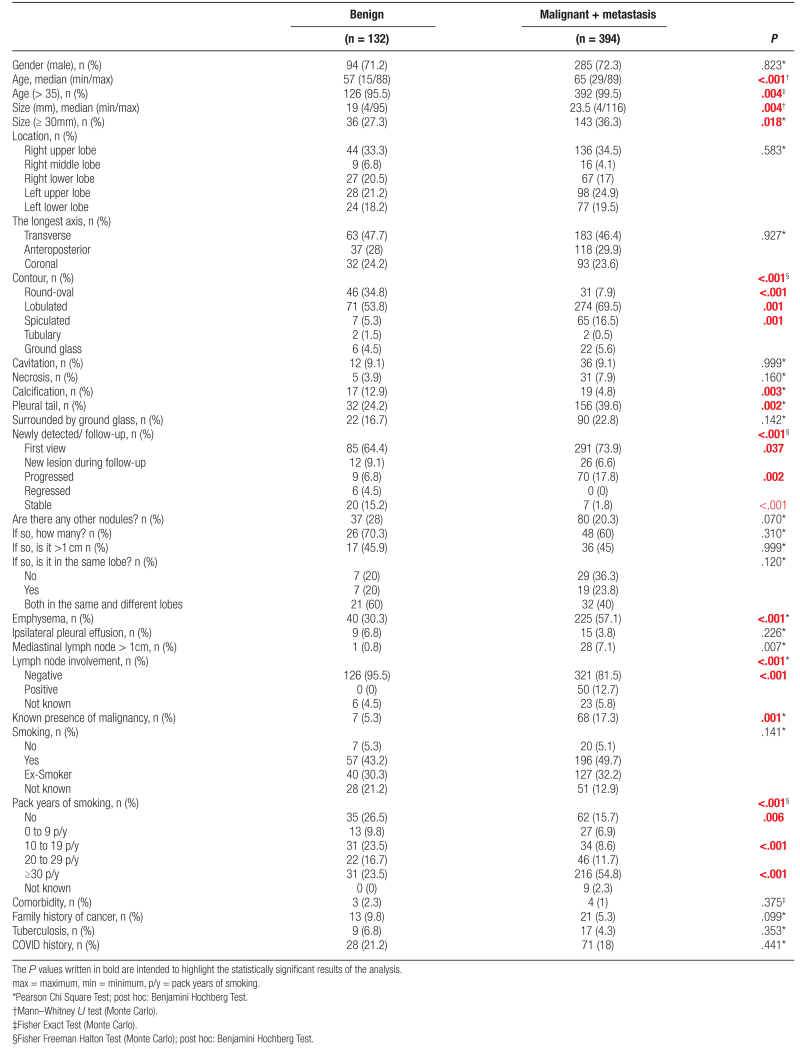
Univariate analysis of demographic characteristics of patients and multiple variables of nodule’s nature.

Similarly, when only nodules (lesions < 30 mm) were included in the analysis, age, lesion contour, calcification content, presence of pleural tail, emphysema and accompanying malignancy indicated a statistically significant relationship for the distinction between benign and malignant nodules (Table 5).

**Table 5:**
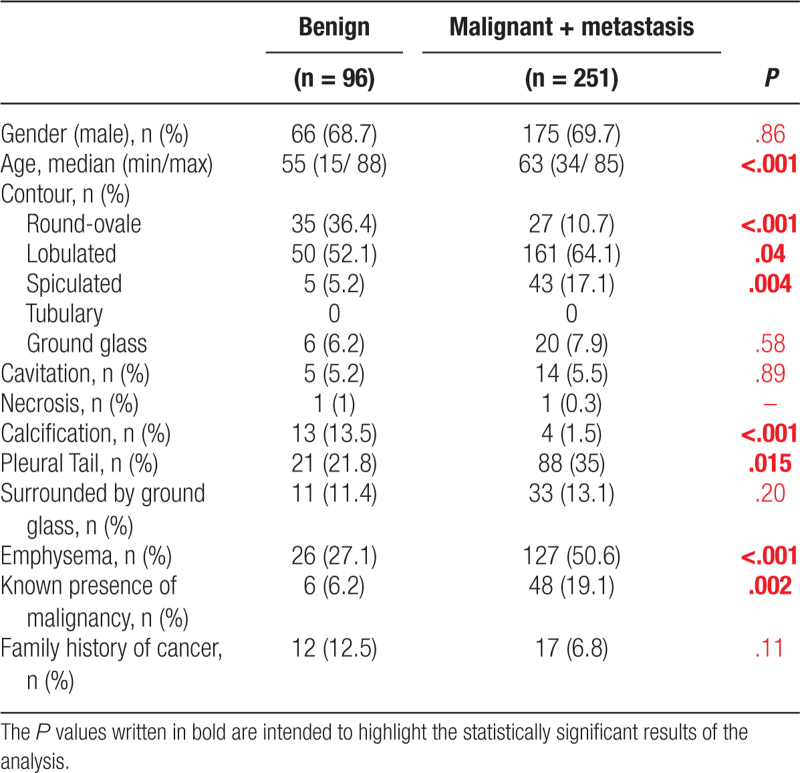
Lung nodules (<3 cm).

## 4. Discussion

When evaluating approaches to pulmonary lesions and their management, several factors must be considered. In general, a lesion of 30 mm or larger is evaluated as malignant until proven otherwise. Therefore, this section will primarily discuss lesions smaller than 30 mm. Lesions smaller than 30 mm are classified as nodules, and the approach to each nodule can vary. Specifically, there are different perspectives and follow-up protocols for nodules smaller than 10 mm in the literature. Currently, the Fleischner Society, BTS, and ACCP provide fundamental guidelines for such cases. However, there are differences among them. For instance, the Fleischner Society considers the average of the long and short axes of the nodule, while the BTS and ACCP guidelines consider the longest axis when developing follow-up and treatment protocols. The Fleischner Society published the latest guidelines on lung nodules in 2017, while the BTS guidelines were published in 2015, and ACCR published their revised version in 2022. In the updated ACCR guideline, it is also suggested that the average of the long and short axes can be taken.^[5]^ All 3 guidelines elaborate on the frequency and duration of follow-up depending on the size and nature of the nodule. We try to carefully follow the recommendations in these guidelines for the patients we treat in our hospital. However, since access to CT devices is much easier today, the rates of lung nodules detected and the number of patients followed radiologically are increasing. Therefore, in our daily practice, we can rarely apply protocols with very short follow-up periods, such as 3 months.

**Figure 5.**
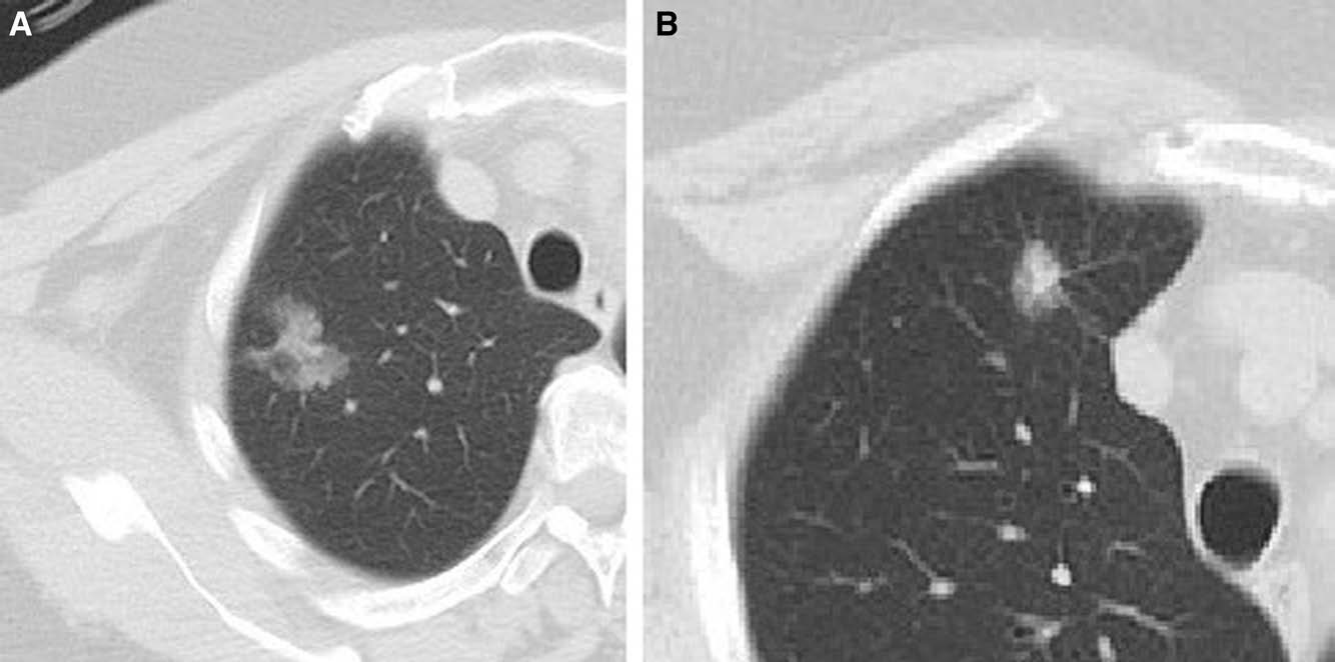
Subsolid nodule examples, (A) Lesion with completely ground-glass density (B) Ground-glass density with a solid component in the center. The histopathological diagnosis of both lesions are adenocarcinoma.

Nodules are classified as solid and subsolid, where subsolid nodules refer to completely ground-glass nodules or those with both a solid component and ground-glass opacity (Fig. 5).^[2–4]^

### 4.1. Age

As age increases, the likelihood of a detected nodule being malignant also rises.^[6]^ The risk of malignancy in nodules under 35 years of age is very low (0.3%). In a study conducted by Byrne et al., 779 patients aged 15 to 34 years were included, excluding those with prior, current, or suspected malignancy. If the nodule was smaller than 10 mm, no malignancy was detected during follow-up. Furthermore, in this patient group, there was no correlation between smoking history, nodule count, or nodule density and malignancy, with nodule size being the only significant factor (*P* = .007).^[7]^ In our study, there were 8 patients younger than 35 years, 2 of whom were diagnosed with malignant nodules, and both nodules were larger than 10 mm (74 and 18 mm).

### 4.2. Gender

The relationship between gender and the nature of pulmonary lesions is not clear. In a study including 7240 patients with incidentally detected pulmonary nodules, the likelihood of malignancy was higher in females compared to males (3.9% vs. 3.4%, respectively).^[8]^ However, in our study, no statistically significant relationship was found between gender and malignancy.

### 4.3. Nodule location

Although some studies have reported that nodules located in the upper lobes are more likely to be malignant compared to those located in non-upper lobes (5.3% vs. 2.6%),^[8]^ we found no significant correlation between the lobe in which the nodule was located and malignancy. Solely, nodules detected in the right middle lobe were more likely to be benign compared to other locations. While the malignancy rate of upper lobe nodules was slightly higher compared to non-upper lobe nodules, it was not statistically significant (74.1% vs. 70%, *P* > .05).

### 4.4. Nodule size and growth rate

The 2 most important radiological features for predicting whether a nodule is malignant are nodule size and growth rate. In subsolid nodules, the presence and size of a solid component are major determinants of malignancy and nodule management.^[9]^ When measuring the size of a subsolid nodule, the solid part is taken into account, and management is planned accordingly.

Nodules smaller than 6 mm have a probability of malignancy of <1%, while those between 6 and 8 mm have a probability of 1% to 2%.^[10]^

In some cases, malignant nodules may be accompanied by satellite benign nodules.^[11]^ When multiple nodules are present, it is not always possible to predict which one is malignant. Although the largest nodule is usually considered malignant, in 20% of cases, the malignant one may not be the largest.^[6]^

Volume doubling time (VDT) is rarely used in clinical practice but is the most sensitive indicator of growth rate, corresponding to a 26% increase in nodule diameter.^[12,13]^ Generally, VDT for malignant pulmonary lesions is up to 400 days, with a VDT < 100 days indicating the highest risk of malignancy. Lesions with VDT >1000 days are malignant in only 1% of cases.^[14]^

However, perifissural nodules may be considered an exception, as these nodules are mostly lymph nodes, and rapid growth does not indicate malignancy.^[15,16]^ Nonetheless, conditions such as spiculation, fissure displacement, and clinical risk factors should be considered.^[2]^

Most lesions in our study were detected on the initial imaging. Among patients with a previous thoracic CT in the hospital Picture Archiving and Communication Systems, there was no statistical difference in distinguishing between benign and malignant lesions in terms of newly developed lesions. However, the risk of malignancy was significantly higher in lesions that showed progression during follow-up, consistent with the literature. While no malignant lesion regressed during follow-up, most stable lesions were benign, as expected. Of the 7 stable but malignant lesions, 6 were adenocarcinomas, and one was a carcinoid tumor. Tumor subtype is also a determining factor for VDT.

### 4.5. Nodule shape

Benign nodules tend to have smooth contours, whereas spiculated contours are largely a characteristic of malignant lesions. Additionally, the presence of a pleural tail is considered a high-risk indicator for malignancy. A lobulated contour may be found in both benign and malignant lesions; however, in our study, we found lobulated contours to be more common in malignant lesions.

Tubular-shaped lesions might suggest benign etiologies such as mucus plugs in ectatic bronchi or vascular structures feeding arteriovenous malformations. However, in our study, 2 out of the 4 tubular lesions were found to be malignant, diagnosed as squamous cell carcinoma and combined large-cell carcinoma.

**Figure 6.**
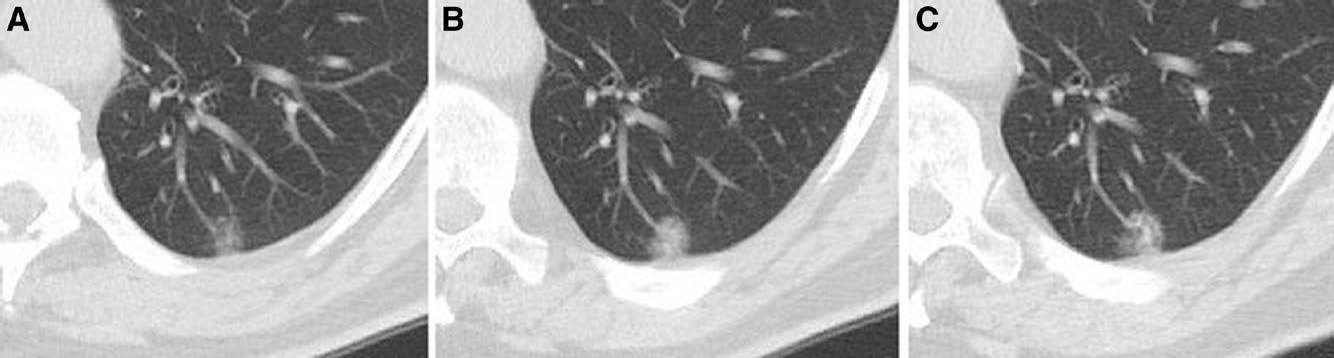
Subsolid nodüle with a completely ground-glass density shows progression and solid component development on follow-up imaging. Histopathological diagnosis is adenocarcinoma.

Subsolid nodules can also cause diagnostic uncertainty. In one study, it was found that subsolid nodules persisting for more than 3 months and larger than 10 mm had a 10% to 50% probability of malignancy. A malignant nodule with a completely ground-glass appearance typically grows slowly (Fig. 6).^[10]^ In our study, benign subsolid nodules were reported in the pathology as organizing pneumonia, foreign body reaction, granulomatous inflammation, interstitial pneumonia, and chronic inflammation. The only benign subsolid nodule that showed progression during follow-up was diagnosed as tuberculosis. Other benign subsolid nodules were either stable or regressed. All malignant subsolid nodules were either stable or progressive during follow-up. This suggests that differentiating between stable subsolid nodules remains challenging, and multiple risk factors, particularly nodule size, must be considered in decision-making.

### 4.6. Cavitation and necrosis

Cavitation and necrosis are often seen in rapidly growing malignant lesions due to the inability of neovascularization to meet the nutritional needs of the rapidly growing lesion. It is more likely to occur in large masses but can also be found in nodules. In our study, 19 out of 347 nodules (5.4%) had cavitation, and 2 nodules (0.5%) had necrosis, with malignancy rates of 73.6% and 50%, respectively.

### 4.7. Calcification and fat content

**Figure 7.**
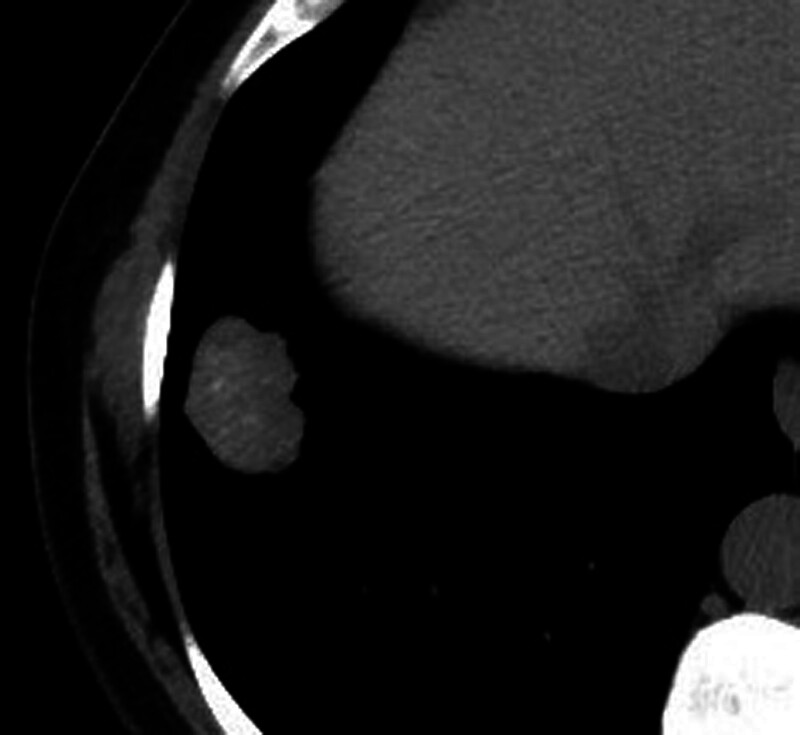
Histopathological diagnosis of the lesion in which punctate calcifications are observed is colon carcinoma metastasis.

The presence of calcification is usually indicative of a benign lesion. However, nodules containing calcification may still carry a risk of malignancy, depending on the calcification pattern. For instance, diffuse, central, or popcorn calcifications suggest benignity, while malignant nodules and metastases may have punctate or eccentric calcifications (Fig. 7). The presence of fat within a nodule is diagnostic for hamartoma and rules out lung cancer.^[17]^

### 4.8. Pleural tail

Fibrotic retractions in the pleura, can be seen in benign etiologies that cause parenchymal destruction, such as tuberculosis, but its relationship with malignant lesions has been known for decades. However, the presence of a pleural tail alone cannot determine the benign or malignant nature of a lesion. Other factors, such as nodule size, must also be considered.^[18]^

### 4.9. Emphysema

The presence of emphysema alone does not determine the nature of a lesion. However, considering that smoking is the most common cause of emphysema and the most significant risk factor for lung cancer, a nodule detected in a patient with emphysema on CT is more likely to be malignant compared to a patient without emphysema.^[19]^ Labaki et al have suggested that emphysema not only provides a conducive environment for malignancy but also promotes tumor growth and aggressiveness.^[20]^ Murakami et al have reported that stage 1 non-small cell lung cancers originating from pulmonary emphysema are associated with higher micro-vessel density, proliferative activity, and postoperative recurrence rates compared to cancers from non-emphysematous lungs.^[21]^ Gonzalez et al have noted that smokers with centrilobular emphysema are at a greater risk compared to those with paraseptal emphysema.^[22]^ In our study, the relationship between emphysema and malignancy was found to be significant in both the entire patient group and the subgroup of patients with nodules, consistent with the literature.

### 4.10. Pleural effusion

The presence of pleural effusion on the same side as the mass may suggest possible malignant effusion. However, when all patient groups were included, our results did not indicate a statistically significant relationship. As the size of the lesion decreases, especially in subcentimetric malignant nodules, malignant effusion is not expected. In our study, all malignant lesions with ipsilateral effusion were larger than 20 mm, with most being larger than 50 mm.

### 4.11. Smoking habit

Smoking is the most well-known risk factor for lung cancer. Quitting smoking reduces the risk of lung cancer; however, even in ex-smokers, the risk remains higher than in those who have never smoked.^[23]^ Passive smoking also increases the risk of lung cancer.^[24]^ Consistent with the literature, in our study, nodules detected in individuals who had never smoked were more likely to be benign, while the highest risk of malignancy was observed in those with a smoking history of ≥30 p/y. There was no increased risk for nodules being malignant with smoking <30 p/y, and nodules detected in patients with 10 to 19 p/y of smoking were more likely to be benign (*P* < .001), which we found to be a surprising result.

### 4.12. History of previous or current malignancy

The risk of developing a second malignancy is high in patients with a known history of malignancy, and lung cancer is the most commonly diagnosed in such cases.^[25]^ The risk of malignancy is high for solitary pulmonary nodules detected in patients who have completed treatment for a primary malignancy.^[26]^ In a study by O’Dwyer et al., it was noted that cancer survivors have a 14% higher risk of developing a second primary malignancy compared to the general population.^[27]^ If a newly developing pulmonary nodule is detected during treatment, the possibility of metastasis must also be considered.

### 4.13. Family history of lung cancer

Having a family history of lung cancer doubles an individual’s risk of developing lung cancer.^[28]^ In our study, no statistically significant difference was found between the nature of the lesion and a family history of cancer, though a lifetime doubled risk differs from the risk of malignancy in a detected nodule at any given point.

### 4.14. Comorbid chronic lung disease

Not only the presence of emphysema but also COPD and IPF increase the risk of lung cancer, thereby increasing the likelihood that a detected lesion is malignant. In one study, a twofold increase in lung cancer risk was reported in COPD diagnosed via pulmonary function tests.^[29]^ Another study suggested that all eligible COPD patients should be screened.^[30]^ Similarly, the risk of lung cancer is increased in pulmonary fibrosis, and most of these patients also have a history of smoking.^[31]^ Lung cancers that develop in IPF patients are mostly squamous cell carcinoma.^[32]^

### 4.15. Tuberculosis and COVID-19

Tuberculosis (TB) infection forms caseating granulomas in the lung parenchyma. Radiologically, these can appear as “tree-in-bud” nodules or multiple scattered nodules. In active infection, cavitary lesions or consolidations may also occur. Diffuse nodules, particularly cavitary lesions with thick and irregular walls, can mimic malignancy. In a study conducted in a region where tuberculosis is endemic, the most commonly diagnosed benign lesion pathologically was granuloma, and the most common malignant lesion was adenocarcinoma. In this study, benign nodules were associated with mostly ≤ 8 mm nodule size, without spiculation, and absence of emphysema.^[33]^ Nodules due to tuberculosis do not have an increased risk of malignancy. However, pulmonary tuberculosis is an independent risk factor for lung cancer, especially in young patients, and patient management must be approached with caution.^[34]^ Qi et al suggest that patients with a history of tuberculosis, especially those with COPD who have never smoked, should be regularly screened or evaluated for lung cancer development.^[30]^

Although the pandemic’s impact has largely diminished today, patients diagnosed with COVID-19 pneumonia with typical lung involvement are still present. Although diagnosis is usually straightforward with well-defined findings, atypical manifestations may sometimes mimic malignancy, and multiple nodules in the parenchyma may be radiologically confused with metastases.^[35]^

### 4.16. Study limitations

This study has some limitations. Firstly, it is a single-center and retrospective study. We did not have the smoking history of all patients. Furthermore, due to the lack of routine dynamic imaging in thoracic CT performed at our hospital, post-contrast density enhancement could not be evaluated.

## 5. Conclusion

Our study aimed to investigate radiological clues for distinguishing between benign and malignant pulmonary lesions, thus facilitating early surgical referral for nodules smaller than 30 mm if progression is observed on thoracic CT, rather than waiting for further progression during follow-up. Accordingly, factors such as older age (>53 years), smoking history (>30 pack years), lesion size (>23 mm), spiculated or lobulated lesion contours, the presence of a pleural tail, emphysema, enlarged mediastinal lymph nodes, and a history of cancer are associated with an increased likelihood of malignancy. The follow-up protocols and other definitions in the 3 main guidelines mentioned above are very valuable, but we believe that applying them, especially in clinics with a high number of patient, is not always feasible. In particular, we recommend histopathological diagnosis instead of radiological follow-up when one of these factors accompanies nodules.

## Author contributions

**Conceptualization:** Ayla Turkar, Elcin Ersoz Kose.

**Data curation:** Ayla Turkar, Elcin Ersoz Kose.

**Investigation:** Ayla Turkar, Elcin Ersoz Kose.

**Methodology:** Ayla Turkar, Elcin Ersoz Kose.

**Project administration:** Ayla Turkar.

**Supervision:** Ayla Turkar, Elcin Ersoz Kose.

**Validation:** Ayla Turkar.

**Visualization:** Ayla Turkar.

**Writing – original draft:** Ayla Turkar.

**Writing – review & editing:** Ayla Turkar, Elcin Ersoz Kose.
